# Role of Selective Histone Deacetylase 6 Inhibitor ACY-1215 in Cancer and Other Human Diseases

**DOI:** 10.3389/fphar.2022.907981

**Published:** 2022-05-16

**Authors:** Jianglei Li, Meihong Yu, Shifeng Fu, Deliang Liu, Yuyong Tan

**Affiliations:** ^1^ Department of Gastroenterology, The Second Xiangya Hospital of Central South University, Changsha, China; ^2^ Research Center of Digestive Disease, Central South University, Changsha, China

**Keywords:** histone deacetylase 6, histone deacetylase inhibitor, ACY-1215, cancer, neurological diseases, inflammatory diseases

## Abstract

The deacetylation process regulated by histone deacetylases (HDACs) plays an important role in human health and diseases. HDAC6 belongs to the Class IIb of HDACs family, which mainly modifies non-histone proteins located in the cytoplasm. HDAC6 plays a key role in tumors, neurological diseases, and inflammatory diseases. Therefore, targeting HDAC6 has become a promising treatment strategy in recent years. ACY-1215 is the first orally available highly selective HDAC6 inhibitor, and its efficacy and therapeutic effects are being continuously verified. This review summarizes the research progress of ACY-1215 in cancer and other human diseases, as well as the underlying mechanism, in order to guide the future clinical trials of ACY-1215 and more in-depth mechanism researches.

## Introduction

Epigenetics, first defined by Professor C.H. Waddington, refers to the heritable modification of gene expression and regulation while does not involve DNA sequence changes, and its related research have been accelerated rapidly in the 21st century (Waddington, 2012; Cavalli and Heard, 2019). Epigenetics includes DNA methylation, histone modification, nucleosome remodeling, and RNA-mediated targeted regulation. They regulate many biological processes that lead to cancer and other human diseases (Dawson and Kouzarides, 2012). Histone acetylation was first identified in 1963 and functionally characterized as a positive regulator of transcription by Vincent Allfrey and colleagues in 1964 (Phillips, 1963; Allfrey et al., 1964). The balance between acetylation and deacetylation is important in regulating gene expression. Histone deacetylases (HDACs) mediate deacetylation, promote the return of chromatin to a suppressed, higher-order structure, which obviously reduces DNA accessibility to the transcription machine. As a result, it will increase transcriptional silencing, and then affect cell fate. Therefore, over acetylation of normally silenced regions or deacetylation of normally active transcription regions may lead to various diseases (Timmermann et al., 2001).

There are 18 subtypes of HDACs in mammals: Class I (HDAC1, HDAC2, HDAC3 and HDAC8), Class II (HDAC4, HDAC5, HDAC6, HDAC7, HDAC9 and HDAC10), Class III (SIRT1, SIRT2, SIRT3, SIRT4, SIRT5, SIRT6 and SIRT7), and Class IV (HDAC11) (de Ruijter et al., 2003). By removing acetyl groups from ε-amino-lysine of proteins (Gallinari et al., 2007), HDACs not only alter transcription, but also promote the establishment or elimination of other post-translational lysine modifications such as methylation and ubiquitination. Biological processes induced by HDACs have a significant impact on human health, and HDACs abnormalities have been documented to play a key role in many human diseases, including cancer, neurological diseases, inflammatory diseases, and heart diseases (Seto and Yoshida, 2014; Zhou et al., 2021).

**FIGURE 1:**
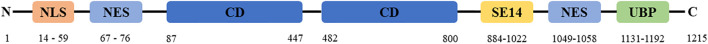
Structure of HDAC6. NLS, nuclear localization sequence. CD, catalytic domains. NES, nuclear export signal. SE14, cytoplasmic retention signal. UBP, ubiquitin-binding zinc finger domain.

In the HDACs family, HDAC6 is the most special as it is the only HDAC with two functional deacetylase domains and a ubiquitin binding zinc finger motif (Verdel et al., 2000; Grozinger et al., 1999; Zhang et al., 2006), containing 1215 amino acid residues (Figure 1). HDAC6 mainly targets proteins located in the cytoplasm. Through the direct deacetylation of tubulin, cortactin and HSP90, or by binding with some chaperonin, HDAC6 regulates the cell response to some important phenomena (Hubbert et al., 2002; Matsuyama et al., 2002; Valenzuela-Fernández et al., 2008; Wang et al., 2018a). Deacetylation of microtubules by HDAC6 is necessary for cell movement, cell cycle regulation, and processing of misfolded proteins (Hubbert et al., 2002; Kawaguchi et al., 2003). The deacetylation of HSP90 by HDAC6 plays an important role in the ubiquitin-proteasome system and protein folding (Yu et al., 2002; Bali et al., 2005; Kovacs et al., 2005). HDAC6 plays an important role in cancer, neurological diseases, inflammatory diseases, and other diseases (Porter et al., 2017; Cosenza and Pozzi, 2018; Ke et al., 2018; Li et al., 2018; LoPresti, 2020; Shen and Kozikowski, 2020). Therefore, inhibitors targeting HDAC6 may be promising treatment modalities.

**FIGURE 2:**
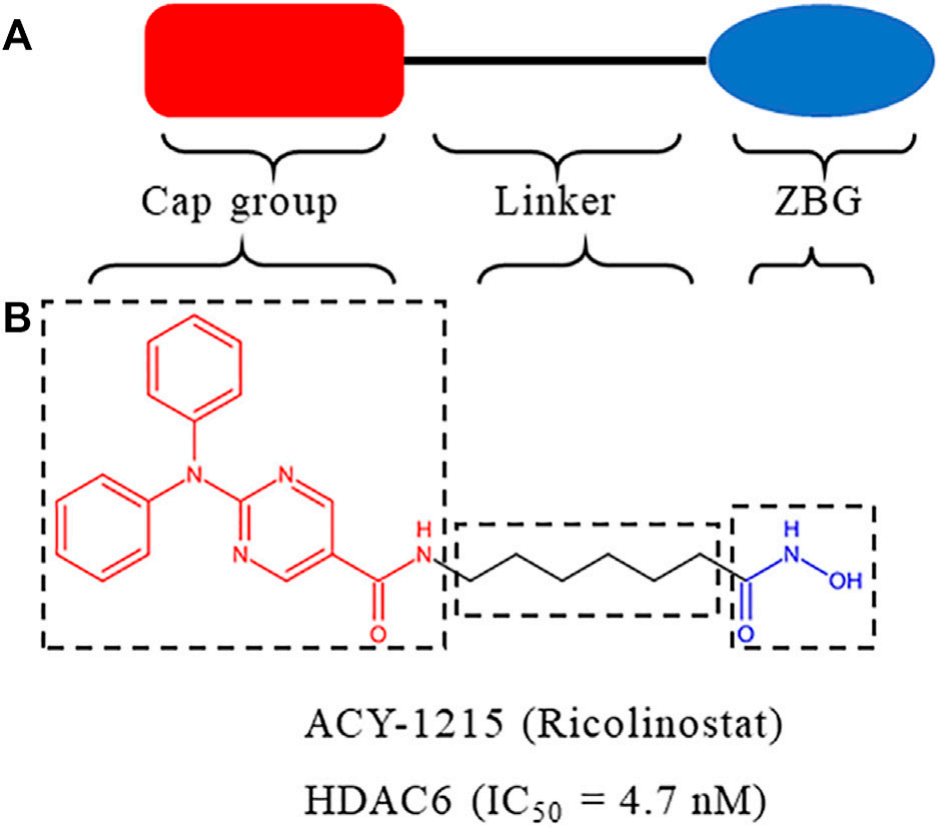
**(A)** Structure of HDACs inhibitor. **(B)** Structure of ACY-1215.

**FIGURE 3:**
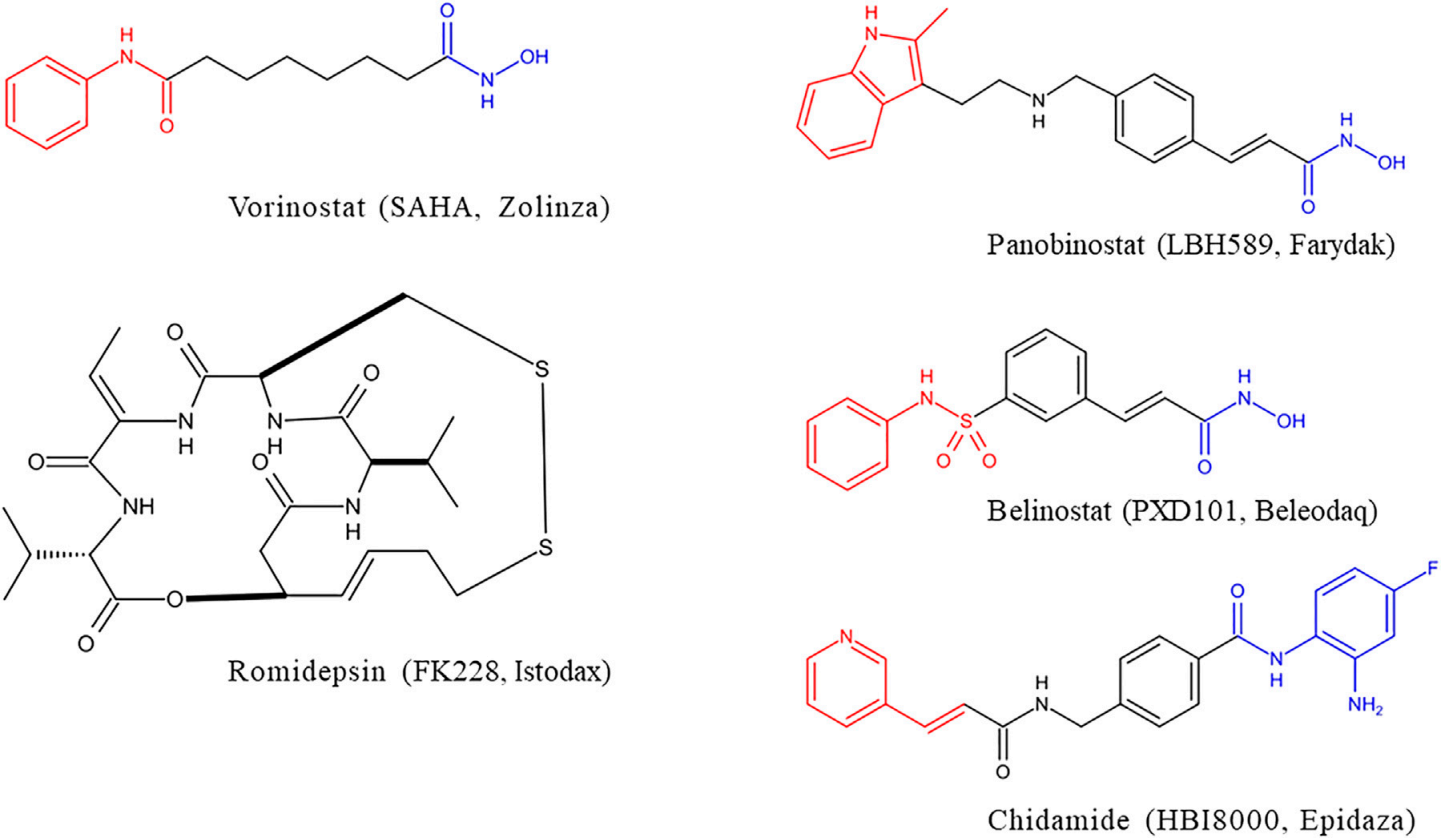
Structure of HDACs inhibitors approved.

HDACs inhibitors are divided into pan-inhibitor and selective inhibitor. HDACs inhibitor has three functional groups. The typical pharmacophore characteristics of HDACs inhibitor is consist of zinc-binding group (ZBG), linker and cap group (Figure 2A). To date, five HDACs inhibitors have been approved: vorinostat (SAHA, Zolinza), romidepsin (FK228, Istodax), panobinostat (LBH589, Farydak), belinostat (PXD101, Beleodaq), and chidamide (HBI8000, Epidaza) (Figure 3) (Whittaker et al., 2010; Duvic and Vu, 2007; Garnock-Jones, 2015; Ning et al., 2012). However, the five HDACs inhibitors are all pan-inhibitors. Due to adverse toxicity such as fatigue, diarrhea, and thrombocytopenia, their clinical application is limited (Falkenberg and Johnstone, 2014; Minucci and Pelicci, 2006). To develop potentially less toxic and more effective treatments, studies on selective HDACs inhibitors are gradually deepening (Zhao et al., 2021; He et al., 2020). Chemical modification of the cap group allows for isomer selective HDACs inhibitors (Krämer et al., 2014). A series of compounds containing urea-based branched linkers with hydroxamate as ZBG have been identified as selective HDAC6 inhibitors (Bergman et al., 2012), such as Tubasatin A, Nexturastat A, ACY-1215 (ricolinostat), ACY-241 (citarinostat), ACY-738, ACY-775, ACY-1083, KA2507, CKD-504,CKD-506 etc (Zhao et al., 2021; Pulya et al., 2021). Among whom, ACY-1215 is a typical representative, having an IC_50_ of 4.7 nM against HDAC6 (Figure 2B). As an effective and the first oral bioavailable selective HDAC6 inhibitor, ACY-1215 is at least 10 times more selective against HDAC6 than other HDACs and is basically non-toxic (Santo et al., 2012; Amengual et al., 2021). ACY-1215 has been studied for long and its importance as anti-cancer agent has already been established through various papers. However, a comprehensive review specifically summarizing its role on cancer and other human diseases is lacking. What’s more, recent studies have found its potential applications in other human diseases and revealed several novel mechanisms. Therefore, we summarized the current study progress of ACY-1215 in cancer and other human diseases.

## ACY-1215 in Cancer

**FIGURE 4:**
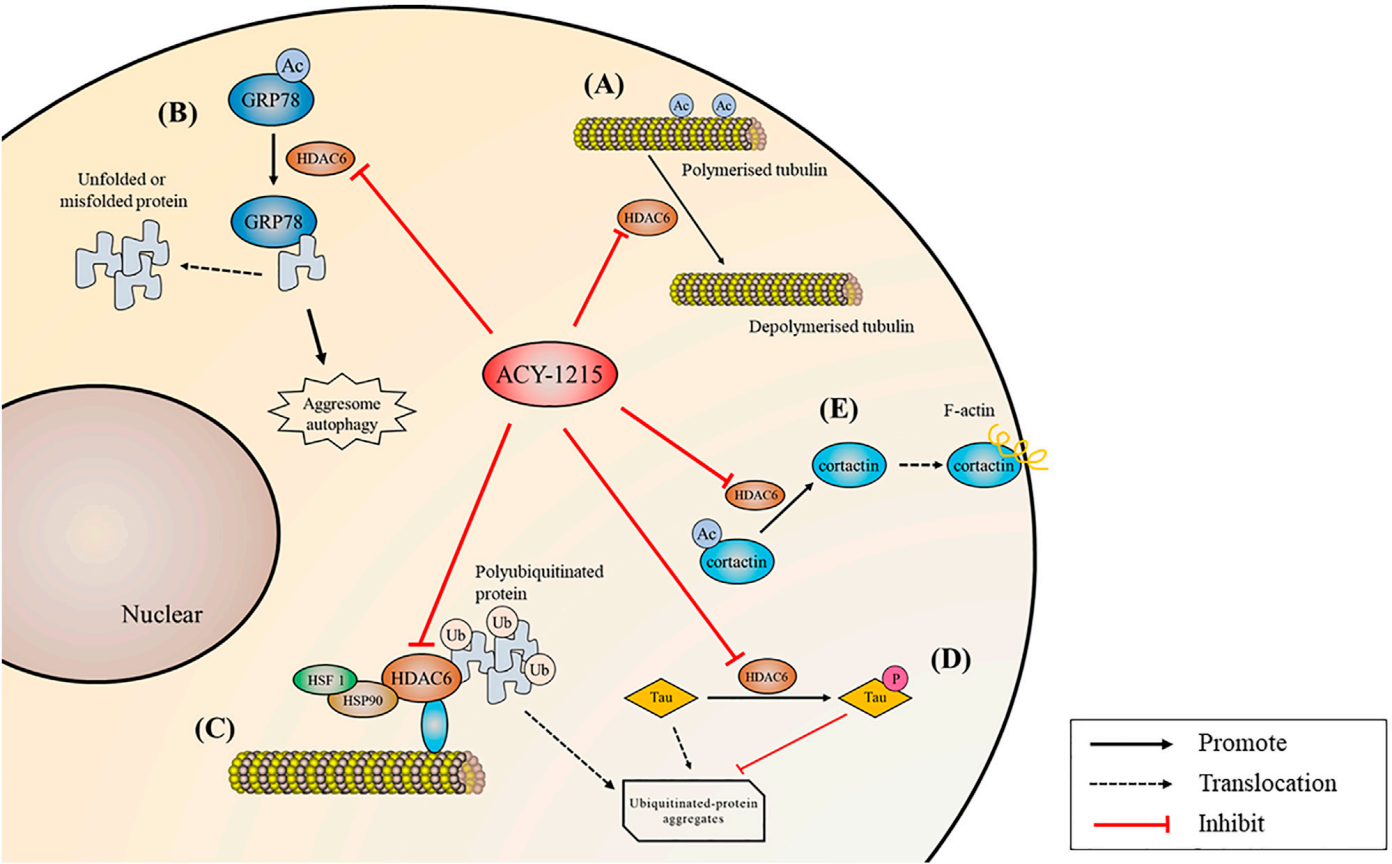
Biological processes regulated by ACY-1215. **(A)** Acetylate tubulin and increase microtubule stability. **(B)** Acetylate GRP78, inhibit misfolded or unfolded protein in the aggregate pathway and inhibit protein degradation. **(C)** Acetylate HSP90 and reduce polyubiquitinated protein transportation. **(D)** Reduce tau hyperphosphorylation and promote clearance. **(E)** Reduce F-actin depended cell migration by deacetylating cortactin.

**FIGURE 5:**
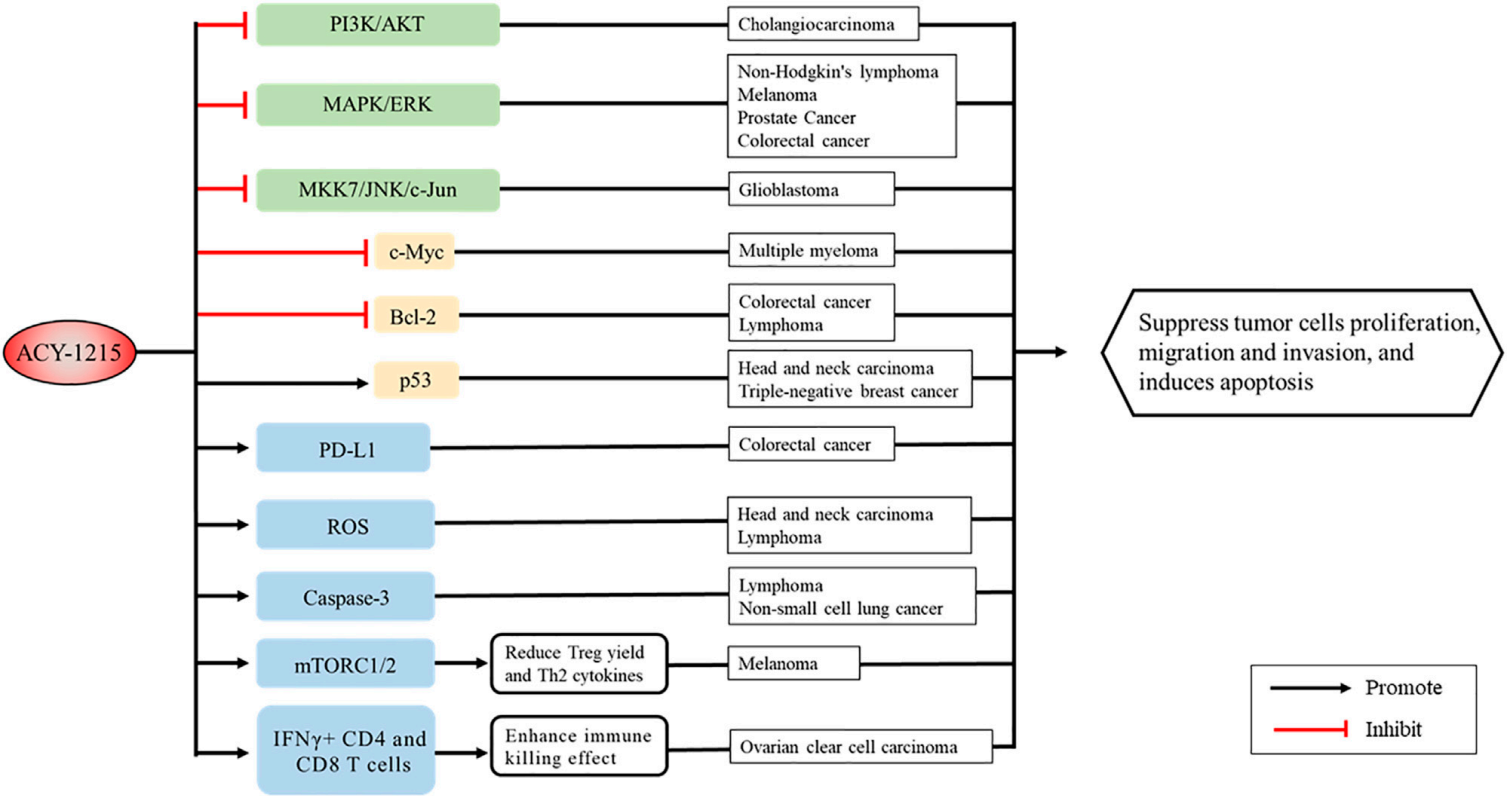
Signaling pathways and proteins in cancer regulated by ACY-1215.

Imbalance of non-histone acetylation is common in human cancers, with changes in the structure or expression of histone acetyltransferases and HDACs occurring in many cancers (Marks et al., 2004). Since the first application of ACY-1215 in multiple myeloma (MM) in 2012 (Santo et al., 2012), ACY-1215 has shown satisfactory efficacy in various tumors. And its molecular mechanism has been gradually revealed (Figures 4, 5).

### Inhibiting Aggresome/Autophagy Pathway and Enhancing Endoplasmic Reticulum Stress

Accumulation of unfolded or misfolded proteins in the endoplasmic reticulum (ER) causes an unfolded protein response, which is a part of ER stress (Senft and Ronai, 2015). Acetylated GRP78 inhibits misfolded or unfolded protein transport along microtubules to centrosomes in the aggregate pathway, inhibiting protein degradation, and then leading to excessive ER stress. Unfolded protein response may induce apoptosis if ER homeostasis cannot be restored (Pfaffenbach and Lee, 2011). MM can be effectively treated with proteasome inhibitors such as bortezomib or carfilzomib, but myeloma cells can overcome proteasome inhibition through alternative aggresome and autophagy pathways to escape from death (Richardson et al., 2017). By deacetylating HSP90, HDAC6 binds and transports polyubiquitinated protein aggregates (Liu et al., 2021). Tereu et al. (Hideshima et al., 2005) found that selective inhibition of HDAC6 increased tubulin acetylation, which in turn inhibited the aggresome pathway, therefore leading to accumulation of polyubiquitinated proteins and apoptosis in MM cells. In 2012, Santo et al. (Santo et al., 2012) applied ACY-1215 for the first time in the treatment of MM, and they found that the combination of low-dose ACY-1215 and bortezomib could produce synergistic anti-MM activity. Compared with pan-HDAC inhibitors, the highly selective ACY-1215 has improved security. Further studies by (Mishima et al., 2015) showed that ACY-1215 inhibited aggresome formation and autophagy caused by carfilzomib induced inhibition of the proteasome pathway, and then induced additional ER stress, thus promoting MM cell apoptosis. In cholangiocarcinoma, reduction of autophagy caused by ACY-1215 decreases proliferation and increases cilia expression (Peixoto et al., 2020). In primary lymphoma, head and neck cancer and melanoma, ACY-1215 and bortezomib have also been demonstrated to have strong synergic effects by dual targeting protein degradation pathways (Amengual et al., 2015; Peng et al., 2017; Hattori et al., 2021).

### Targeting Cell Signaling Pathways and Related Gene Expression

ACY-1215 has also been demonstrated to be an important regulator of apoptosis pathways in tumors. ACY-1215 inhibits cell proliferation and promotes apoptosis by targeting MAPK/ERK, PI3K/AKT and other cell signaling pathways. In non-Hodgkin’s lymphoma, ACY-1215 causes inactivation of AKT and ERK1/2, leading to increased DNA damage and ultimately tumor cells death (Lee et al., 2019a). In melanoma cells, ACY-1215 accelerates cell death by inhibiting ERK activation (Peng et al., 2017; Sundaramurthi et al., 2022). In head and neck carcinoma cell, the suppression of p-Chk1 activity caused by ACY-1215 leads to synergistically enhanced apoptosis via mitotic catastrophe in a p53-dependent manner (Miyake et al., 2022). Enhanced transcriptional activity of p53 by ACY-1215 is also found in triple-negative breast cancer (Cao et al., 2022). In cholangiocarcinoma, ACY-1215 suppresses GRP78 translocation to the cell surface via PI3K/AKT pathway, which inhibits proliferation and promotes apoptosis (Kim et al., 2022). Growth inhibition has also been observed in colon cancer cells, prostate cancer cells, glioma cells and gallbladder cancer cells (Tan et al., 2019; Corno et al., 2020; Huang et al., 2020; Ruan et al., 2021). Besides, ACY-1215 in combination with immunosuppressant IMiD (Hideshima et al., 2015) and inhibitor JQ1 (Carew et al., 2019), a member of the Bromine domain and extra terminal protein family, down-regulated proto-oncogene c-Myc expression and induced co-cytotoxicity in MM.

### Inhibiting Cell Cycle, Cell Migration and Motility

Abnormal cell cycle and strong migration ability of tumor cells lead to rapid proliferation and high degree of malignancies. The change of microtubule dynamics can lead to cell cycle stagnation. ACY-1215 induces apoptosis and G0/G1 cell cycle arrest by increasing tubulin acetylation in melanoma cells (Wang et al., 2018b). In non-small cell carcinoma cell lines A549, LL2, and H1299, inhibition of HDAC6 by ACY-1215 leads to G2 phase arrest and increased apoptosis (Deskin et al., 2020). In ARID1A-deficient endometrial carcinoma, the G2/M cell cycle checkpoint and ATM/ATR-mediated DNA damage checkpoints is disrupted, while the migratory and invasive phenotype can be reversed by ACY-1215 (Megino-Luque et al., 2022). In triple-negative breast cancer, ACY-1215 results in G1 cell cycle arrest and apoptosis (Cao et al., 2022), and enhances the anti-tumor effect of eribulin through tubulin acetylation (Oba et al., 2021). Moreover, F-actin depended cell migration is also reduced when cortactin deacetylation is inhibited (Li et al., 2018). ACY-1215 inhibits the proliferation and migration of high-grade serous ovarian cancer cells and tektin4-deficient triple-negative breast cancer cells (Ali et al., 2020; Ge et al., 2021).

### Revitalizing the Function of Immune Cells, Promoting the Killing Ability Against Cancer Cells

Immunotherapy is an important part of cancer therapy (van den Bulk et al., 2018; Yang, 2015). Programmed death ligand 1 (PD-L1) expression is significantly increased in ACY-1215 combination therapy in colorectal cancer cells (Ryu et al., 2018). (Lee et al., 2018) further verified that the combination of ACY-1215 and oxaliplatin could not only induce the synergistic upregulation of PD-L1, but also decreased the level of Bcl-2 protein and some other kinase. In ovarian clear cell carcinoma, ACY-1215 was found to activate CD4 and CD8 T cells and increase IFNγ+ CD4 and CD8 T cells, as a result enhancing the immune killing effect (Fukumoto et al., 2019). In melanoma patients, ACY-1215 downregulates mTORC1/2 signaling, reduces yield of Treg and production of Th2 cytokines, thereby, altering T-cell function (Laino et al., 2019). The combination of ACY-1215 and JQ1 in the treatment of xenograft tumors derived from human and mouse small-cell lung cancer cell lines showed significant tumor growth inhibition by provoking NK-cell-mediated immunity (Liu et al., 2018).

The antitumor effects of HDAC6 inhibitors were also demonstrated in other selective HDAC6 inhibitors, such as ACY-241 (Ray et al., 2018; Cosenza et al., 2020; Awad et al., 2021; Park et al., 2021) and KA2507 (Tsimberidou et al., 2021).

## ACY-1215 in Neurological Diseases

In addition to bind and transport polyubiquitinated proteins for aggregation, HDAC6 can also regulate domain receptors for cytoskeletal proteins such as tau, IIp45 (invasion inhibitory protein 45) and EGFR (epidermal growth factor receptor) through protein-protein interactions (Pulya et al., 2021). HDAC6-mediated acetylation of multiple non-histones is associated with different functions including intracellular transport, neurotransmitter release, and aggregation formation (Chen et al., 2010; Kalinski et al., 2019). Dysregulation of HDAC6 results in alterations in excitatory-inhibitory equilibrium, synaptic transmission, memory, and protein processing. HDAC6 inhibitors regulate a variety of events including growth cone function, synaptic plasticity, transport and autophagosome degradation (LoPresti, 2020). Inhibition of HDAC6 restores α -tubulin acetylation and mitochondrial transport (Perry et al., 2017). In addition, HDAC6 inhibitors promote degradation of protein aggregates and protection from neuronal oxidative stress (Wang et al., 2019; Zeb et al., 2019). Therefore, ACY-1215 may play a vital role in neurodegeneration and peripheral neuropathy.

### Decreasing Levels of Amyloid Beta Load and Tau Hyperphosphorylation

Neurodegenerative diseases are a kind of nervous system diseases closely related to aging (Hou et al., 2019). Low acetylation is present during neurodegeneration (Sharma et al., 2019). HDAC6 may not only lead to deterioration of learning and memory, but also increase Aβ and tau phosphorylation levels (Liu et al., 2020). Extracellular aggregation of Aβ plaques and intracellular neurofibrillary tangles composed of hyperphosphorylated tau protein in the human cortex and limbic regions contribute to the development of Alzheimer’s disease (AD). Tau usually binds to and stabilizes microtubules. But in AD and related neurodegenerative diseases, significantly increased HDAC6 reduces tubulin acetylation, as a result, tau is hyperphosphorylated and aggregates into neurofibrillary tangles, which eventually leads to neuron loss, synaptic dysfunction, and cognitive decline (Hempen and Brion, 1996; Yan, 2014; Tiwari et al., 2019; Li et al., 2021). (Zhang et al., 2014) found that ACY-1215 effectively reduced the behavioral defects of AD mice by reducing Aβ deposition and tau hyperphosphorylation, as well as promoting autophagy clearance. (Mao et al., 2017) further found in *drosophila* that by increasing the acetylation of tubulin, ACY-1215 could rescue microtubules defects and neuromuscular junction growth anomalies caused by tau overexpression. The same results were also noticed in another selective HDAC6 inhibitor CKD-504 (Choi et al., 2020).

### Improving Mitochondrial Function and Axon Transport Defects

Cognitive impairment of the nervous system is directly related to axon damage. (Wang et al., 2019) found that by increasing tubulin acetylation, ACY-1215 decreased mitochondrial transport and mitochondrial dysfunction and increased synaptic density, thus ameliorating cisplatin-induced brain damage in mice. The same conclusion was found in hippocampus mitochondria (Ma et al., 2018). In HIV-positive patients, binding of GP120 to neuronal microtubules and reduced tubulin acetylation levels decreased the rate of axon transport of brain-derived neurotrophic factor. Wenzel et al. showed that ACY-1215 blocked GP120-mediated tubulin deacetylation and axon transport reduction (Avdoshina et al., 2017; Wenzel et al., 2019).

In addition to affecting cognitive function, axon transport disorders are associated with peripheral neuropathy (Pareyson et al., 2015; Prior et al., 2018). Peripheral neuropathy is a chronic, debilitating disease that involves peripheral nerve damage in varies diseases such as Charcot-Marie-Tooth (CMT) disease, chemotherapy neurotoxicity, mitochondrial disease, and diabetes (Colloca et al., 2017). Studies have found that mechanical abnormal pain occurs due to mitochondrial damage in neurons (Ma et al., 2019). ACY-1215 can effectively reverse cisplatin-induced mechanical abnormal pain, and the effect still exists 1 week after completion of treatment (Krukowski et al., 2017). CMT2 is a non-demyelinating axonal disease characterized by muscle weakness and atrophy (Morena et al., 2019). What’s more, ACY-1215 ameliorates mitochondrial transport deficits by increasing tubulin acetylation, which in turn rescue axon transport deficits and then reverse motor and sensory deficits in a mouse model for mutant “small heat shock protein B1”-induced CMT2 at both behavioral and electrophysiological levels (Benoy et al., 2017). The effect of ACY-1215 on CMT has also been demonstrated in CKD-504 (Ha et al., 2020; Smith et al., 2022).

## ACY-1215 in Inflammatory Diseases

**FIGURE 6:**
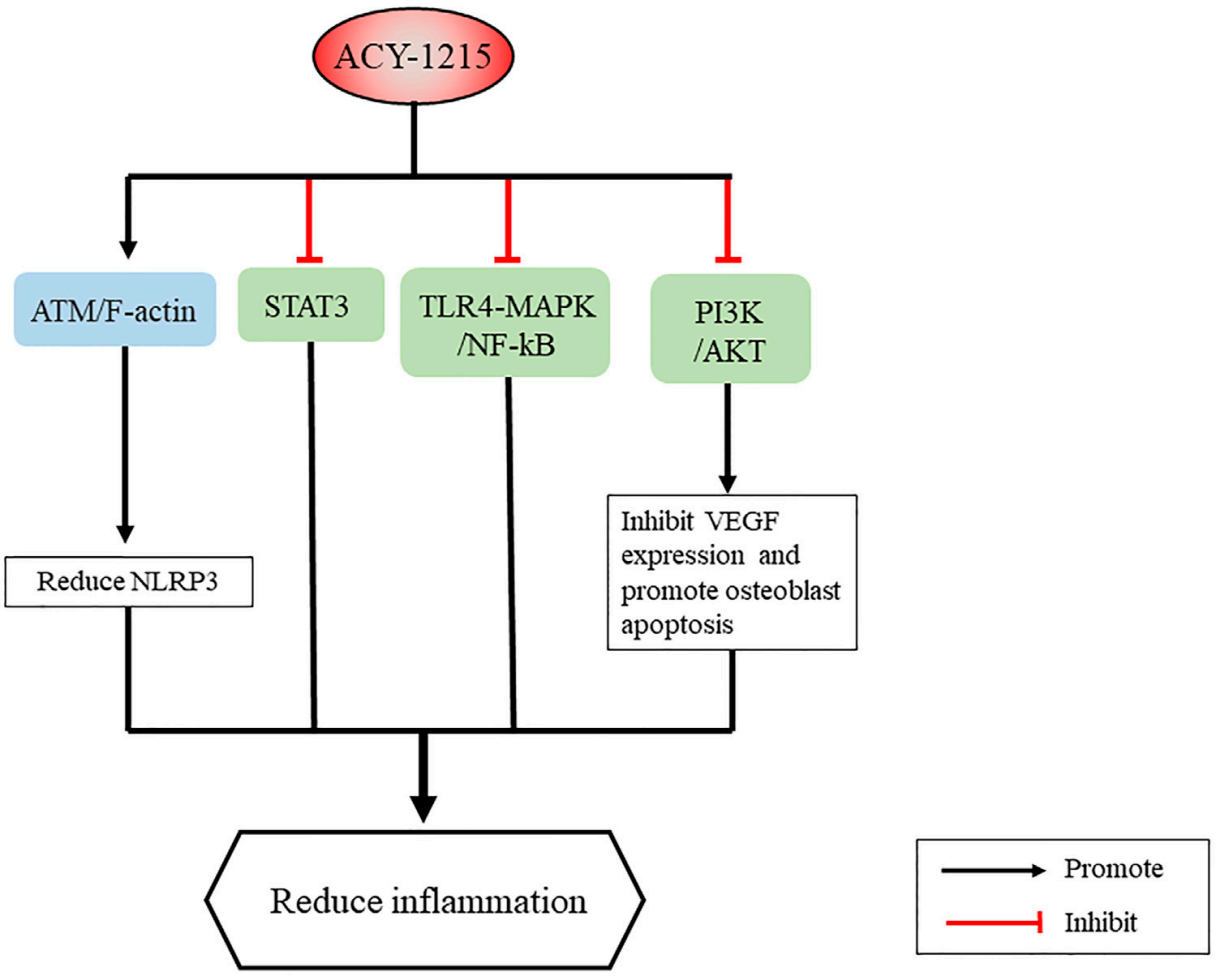
Signaling pathways and proteins in inflammation regulated by ACY-1215.

Generally, inflammation is a defensive response of lesion present in living tissue (Shi and Pamer, 2011). However, dysregulated, or excessive inflammation can be harmful. Through regulating cell signaling pathways, inflammatory cytokines, and inflammatory cells (Ran and Zhou, 2019; Lee et al., 2020), HDAC6 inhibitors have great potential as a treatment for inflammatory diseases, including rheumatoid arthritis (Oh et al., 2017), inflammatory bowel disease (Lu et al., 2016; Do et al., 2017), and respiratory inflammation (Ren et al., 2016). In addition, ACY-1215 has also shown to promising results in acute liver failure (ALF), osteoarthritis, and skin inflammation (Figure 6).

Gong et al. (Jiao et al., 2017; Zhang et al., 2018; Chen et al., 2021) applied ACY-1215 to a mouse model of ALF. They showed that ACY-1215 improved liver tissue damage and liver function in ALF, reduced the expression level of inflammatory factor TNFα-mRNA and NF-κB-P65 protein, and reduced NLRP3 inflammasome through regulation of ATM/F-actin signaling pathway. *In vitro*, ACY-1215 mitigated LPS-induced macrophage inflammation via the TLR4-MAPK/NF-κB pathway (Zhang et al., 2019a). Furthermore, ACY-1215 can also activate AMPK signaling pathway, enhance autophagy, regulate mitochondrial mediated oxidative stress, improve glucose metabolism and lipid metabolism, and thereby reduce apoptosis and inflammatory response in ALF (Zhang et al., 2019b; Chen et al., 2019; Chen et al., 2020a; Wang et al., 2021). In osteoarthritis, ACY-1215 inhibits the STAT3 and NF-κB pathway in chondrocytes leading to anti-inflammatory and chondroprotective effects (Cheng et al., 2019), as well as inhibits VEGF expression through the PI3K/AKT pathway and then promotes osteoblast apoptosis (Li et al., 2019). In addition, ACY-1215 improves neuropathic pain by blocking MyD88-dependent pro-inflammatory pathways (Chen et al., 2022). HDAC6 inhibitors inhibit inflammation by inhibiting NF-κB signaling, reducing the production of various pro-inflammatory cytokines and chemokines, and inhibiting the inflammatory response of monocytes/macrophages, which was also demonstrated in CKD-506 (Choi et al., 2018; Lee et al., 2020; Park et al., 2020; Park et al., 2021). Another interesting finding was that ACY-1215 inhibited the function of impaired CD8 T cells during skin inflammation, preventing the development of contact hypersensitivity and graft-versus-host disease like-diseases *in vivo* by regulating CD8 T cell activation and function (Tsuji et al., 2015).

## ACY-1215 in Other Diseases

Because of ACY-1215’s involvement of various pathway, attempts in varies disease models have yielded inspiring results. In polycystic liver disease, ACY-1215 diminished liver cyst development and fibrosis by reducing cholangiocyte proliferation and cyst growth both *in vitro* and *in vivo* (Gradilone et al., 2014). ACY-1215 with pasireotide synergistically reduced cyst growth and increased length of primary cilia *in vivo*, and decreased cell proliferation and inhibited cAMP levels *in vitro* (Lorenzo Pisarello et al., 2018). In a mouse model of polycystic kidney disease, ACY-1215 treatment reduced cAMP and cyst growth (Yanda et al., 2017). And ACY-1215 mitigated renal fibrosis by suppressing transforming growth factor-β1 and epidermal growth factor receptor signaling pathways in obstructive nephropathy (Chen et al., 2020b). In glucocorticoid-induced osteoporosis, ACY-1215 reverses dexamethasone-induced inhibition of osteoblast proliferation and differentiation (Wang et al., 2020). In rats with cardiac ischemia-reperfusion injury, ACY-1215 might reduce infarct size through modulating hypoxia inducible factor-1α expression (Lin et al., 2020). Although this part of the study did not have a more in-depth mechanism discussion, it also provided a new treatment idea for the diseases.

## Future Perspective

HDAC6 regulates various biological processes involved in proteasome degradation, cell migration, microtubule dynamics, apoptosis, and axon growth, and it also participates in a variety of signaling pathways in pathological responses to diseases. Targeting the above biological process by inhibiting the functions and activities of HDAC6 are well studied in different cancers, neurodegenerative diseases, epigenetic rare diseases, and inflammatory diseases. To date, many selective HDAC6 inhibitors have been have been reported in preclinical studies and have entered clinical trials (Tables 1, 2). However, except for ACY-1215, the present application range of other HDAC6 inhibitors is limited. Studies on ACY-241 (Ray et al., 2018; Cosenza et al., 2020; Awad et al., 2021; Park et al., 2021) and KA2507 (Tsimberidou et al., 2021) mainly focused on tumors, CKD-504 (Choi et al., 2020; Ha et al., 2020; Jeong et al., 2022; Smith et al., 2022) focused on neurological diseases, and CKD-506 (Choi et al., 2018; Park et al., 2020; Bae et al., 2021) focused on inflammatory diseases. Although ACY-241 and KA2507 show higher selectivity over ACY-1215 on HDAC6, its studies on other diseases needs further research (Table 3).

**TABLE 1:** ACY-1215 in different phases of clinical trials.

NCT number	Condition or disease	Registration date	Status	Phase	Dosage of ACY-1215	Combination drug
NCT02091063	Lymphoid Malignancies	March 19, 2014	Completed	Ⅰ/Ⅱ	Phase Ⅰ: 160 mg QD or 160 mg BID	N/A
					Phase Ⅱ: 160 mg BID	
NCT01323751	Multiple Myeloma	March 28, 2011	Completed	Ⅰ/Ⅱ	Phase Ⅰ: 40, 80, 160, 240 mg QD, or 160 mg BID	Bortezomib and dexamethasone
					Phase Ⅱ: 160 mg QD	
NCT01997840	Multiple Myeloma	November 28, 2013	Active, not recruiting	Ⅰ/Ⅱ	160 mg QD	Pomalidomide and dexamethasone
NCT02632071	Metastatic Breast Cancer	December 16, 2015	Completed	Ⅰ	80, 120, 180, or 240 mg QD	Paclitaxel
NCT02189343	Multiple Myeloma	July 14, 2014	Completed	Ⅰ	N/A	Pomalidomide and dexamethasone
NCT02787369	Chronic Lymphoid Leukemia	June 1, 2016	Active, not recruiting	Ⅰ	N/A	Ibrutinib or idelalisib
NCT01583283	Multiple Myeloma	April 24, 2012	Completed	Ⅰ	Ranging from 40 to 480 mg QD	Lenalidomide and dexamethasone
NCT02088398	Healthy Subjects	March 17, 2014	Completed	Ⅰ	120 or 160 mg QD	N/A
NCT03176472	Painful Diabetic Peripheral Neuropathy	June 5, 2017	Recruiting	Ⅱ	120 mg QD	N/A
NCT05193851	Peripheral Nervous System Diseases	January 18, 2022	Recruiting	Ⅰ	N/A	N/A
NCT05229042	Chemotherapy-Induced Peripheral Neuropathy	February 8, 2022	Not yet recruiting	Ⅰ	N/A	N/A
NCT02661815	Gynecological Cancer	January 25, 2016	Terminated	Ⅰb	N/A	Paclitaxel and/or Bevacizumab

N/A: not available.

**TABLE 2:** Other HDAC6 inhibitors in clinical trials.

Inhibitor	NCT number	Condition or disease	Registration date	Status	Phase	Dosage	Combination drug
ACY-241	NCT02551185	Advanced Solid Tumors	September 16, 2015	Completed	Ⅰ	180, 360, or 480 mg QD	Paclitaxel
ACY-241	NCT02635061	Non-Small Cell Lung Cancer	December 18, 2015	Active, not recruiting	Ⅰ	180, 360, or 480 mg QD	Nivolumab
ACY-241	NCT02400242	Multiple Myeloma	March 27, 2015	Active, not recruiting	Ⅰ	Ranging from 180 to 480 mg QD	Pomalidomide and dexamethasone
ACY-241	NCT02935790	Malignant Melanoma	October 18, 2016	Completed	Ⅰ	N/A	Ipilimumab and nivolumab
KA2507	NCT03008018	Solid Tumors	January 2, 2017	Completed	Ⅰ	50, 100, 200 mg QD, or 200, 400, 800 mg BID	N/A
CKD-504	NCT03713892	Huntington Disease	October 22, 2018	Recruiting	Ⅰ	N/A	N/A
CKD-506	NCT05238948	Healthy Subjects	February 14, 2022	Recruiting	Ⅰ	N/A	Midazolam
CKD-506	NCT04204603	Rheumatoid Arthritis	December 19, 2019	Completed	Ⅱ	N/A	N/A
CKD-510	NCT04746287	Healthy Subjects	February 9, 2021	Active, not recruiting	Ⅰ	N/A	N/A

N/A: not available.

**TABLE 3:** Inhibition of HDAC6 inhibitors on HDACs.

	IC50, nM					
	ACY-1215 (Santo et al., 2012)	ACY-241 (Huang et al., 2017)	KA2507 (Tsimberidou et al., 2021)		CKD-504 (Choi et al., 2020)	CKD-506 (Choi et al., 2018)
HDAC1	58	35	9895		>10,000	>2000
HDAC2	48	45	>10,000		>10,000	>2000
HDAC3	51	46	>10,000		>10,000	N/A
HDAC4	7000	>20,000	9613		>10,000	N/A
HDAC5	5000	>20,000	1997		>10,000	N/A
HDAC6	4.7	2.6	2.5		46	5
HDAC7	1400	7300	2333		>10,000	>2000
HDAC8	100	137	621		6600	>2000
HDAC9	>10,000	>20,000	5648		>10,000	N/A
HDAC11	>10,000	N/A	>10,000		>10,000	N/A
Sirtuin 1	>10,000	N/A	N/A		N/A	N/A
Sirtuin 2	>10,000	N/A	N/A		N/A	N/A

N/A: not available.

It has been more than 10 years since the discovery and application of ACY-1215. Currently, there are more than 10 phase I/II clinical trials related to ACY-1215. The existing trial results show that at the recommended dose of ACY-1215 of 160 mg daily, the combination with bortezomib/lenalidomide and dexamethasone of MM therapy has a higher treatment response and without adverse events (Yee et al., 2016; Vogl et al., 2017). The safety and efficacy of ACY-1215 in patients with recurrent and refractory lymphatic malignancies were also demonstrated (Amengual et al., 2021). Meanwhile, ACY-1215 could have meaningful clinical impact on preventing or attenuating taxane-induced peripheral neuropathy (Lee et al., 2019b). While, there are currently no phase III clinical trials of ACY-1215 ongoing. Therefore, phase III clinical trials of the above or clinical trials on the application of ACY-1215 on other diseases need further investigation.

At present, ACY-1215 has achieved significant therapeutic effects among various diseases in cell and animal models, and the involved pathway is relatively clear. However, there are still some objective problems that cannot be ignored. On the one hand, the limitations of the disease model itself lead to the distance gap from laboratory to clinic, and on the other hand, the safety and efficacy of ACY-1215 still need more clinical trials to prove. The latest researches also showed that ACY-1215 could improve the developmental competence of somatic cell nuclear transfer embryos (Gao et al., 2022) and promote the generation of megakaryocyte progenitors (Jiang et al., 2022).

In the present review, we summarized the research progress of ACY-1215 in cancer and other human diseases, as well as its related mechanisms. This review will guide researchers to further explore the clinical application of ACY-1215 to various diseases and further reveal its underlying molecular mechanisms.
