# Corrigendum: Role of selective histone deacetylase 6 inhibitor ACY-1215 in cancer and other human diseases

**DOI:** 10.3389/fphar.2022.1117936

**Published:** 2022-12-14

**Authors:** Jianglei Li, Meihong Yu, Shifeng Fu, Deliang Liu, Yuyong Tan

**Affiliations:** ^1^ Department of Gastroenterology, The Second Xiangya Hospital of Central South University, Changsha, China; ^2^ Research Center of Digestive Disease, Central South University, Changsha, China

**Keywords:** histone deacetylase 6, histone deacetylase inhibitor, ACY-1215, cancer, neurological diseases, inflammatory diseases

In the published article, there was an error in the **Funding** statement. “This work was supported by Scientific research project of Hunan Provincial Health Commission (No. 202103030980) and Graduate Research Innovation Project of Central South University.” The correct Funding statement appears below.

